# School‐based mental health and psychosocial support interventions for children and adolescents with developmental disabilities in low‐ and middle‐income countries: A systematic review

**DOI:** 10.1111/tmi.70000

**Published:** 2025-06-24

**Authors:** Maria Jose Alpuche De Lille, Renata Teixeira da Silva, Tracey Smythe

**Affiliations:** ^1^ London School of Hygiene & Tropical Medicine London UK; ^2^ Department of Health Service and Population Research, Institute of Psychiatry, Psychology and Neuroscience King's College London London UK; ^3^ Department of Health and Rehabilitation Sciences, Division of Physiotherapy Stellenbosch University Cape Town South Africa

**Keywords:** children and adolescents mental health, developmental disabilities, intellectual disabilities, low and middle‐income countries, Mental Health and Psychosocial Support (MHPSS), neurodevelopmental disorders, school‐based interventions

## Abstract

**Objectives:**

To identify and evaluate the characteristics and reported effects of school‐based mental health and psychosocial support interventions targeting children and adolescents with neurodevelopmental disorders (NDDs) in low‐ and middle‐income countries, as well as those involving their parents, teachers or peers.

**Methods:**

A systematic search of MEDLINE, EMBASE, ERIC, Global Health and PsycINFO was conducted in October 2024. Eligible studies included randomised controlled trials, quasi‐experimental and qualitative research on school‐based interventions in low‐ and middle‐income countries for children and adolescents with NDDs (including attention deficit hyperactivity disorder [ADHD], autism, intellectual disabilities, epilepsy, cerebral palsy and foetal alcohol syndrome), as well as those involving their caregivers, teachers or peers. Only studies published in English, Spanish or Portuguese were included. A narrative synthesis was performed.

**Results:**

A total of 2158 titles were screened, with 29 studies from 13 countries included. Most studies used a quasi‐experimental design (*n* = 19, 66%). Nearly half focused on children and adolescents with NDDs only (*n* = 14, 48%), with intellectual disabilities being the most targeted condition (*n* = 12, 34%), followed by autism (*n* = 8, 23%) and ADHD (*n* = 8, 23%). Intervention strategies included multimodal approaches (*n* = 6, 21%) and educational workshops (*n* = 6, 21%). Targeted outcomes were social skills (*n* = 7, 16%) and knowledge attitudes and practice (*n* = 5, 12%). Lifelong learning (*n* = 11, 33%) and educational system‐strengthening interventions (*n* = 10, 31%) were the primary content areas. The majority of studies exhibited a moderate to high risk of bias.

**Conclusions:**

Schools offer strategic platforms for delivering mental health and psychosocial support interventions to children and adolescents with NDDs in low‐ and middle‐income countries, involving families, teachers and peers. While improvements in social skills and knowledge, attitudes and practices were reported, heterogeneity and methodological limitations constrain the generalisability of findings. Future research should address long‐term impacts and expand to underrepresented conditions.

## 1 INTRODUCTION

Children with disabilities include ‘those who have long‐term physical, mental, intellectual or sensory impairments which in interaction with various barriers may hinder their full and effective participation in society on an equal basis with others.’ [1] Developmental disabilities are a diverse group of conditions that affect the developing nervous system, often leading to impairments in motor, cognitive, language, behavioural and/or sensory functioning. Developmental disabilities are also often defined as chronic physical, cognitive, speech or language, psychological or self‐care conditions that originate during childhood; are likely to continue indefinitely and consequently require additional coordinated services and support during this time; and represent a subset of conditions that affect children with special health care needs [2, 3, 4]. When these impairments intersect with environmental barriers and contextual factors, they can hinder full participation in society [5, 6]. There are approximately 316.8 million children and adolescents with developmental conditions [6]. Additionally, 95% of children with developmental disabilities under the age of five live in low‐ and middle‐income countries (LMICs), where access to adequate health and educational resources is often constrained [7], aggravating disability. Within the spectrum of developmental disabilities, neurodevelopmental disorders (NDDs) include attention deficit hyperactivity disorder (ADHD), autism, cerebral palsy, epilepsy, foetal alcohol spectrum disorders and idiopathic developmental intellectual disability (ID). Other developmental disabilities include sensory impairments such as congenital hearing or visual impairment and spina bifida. However, NDDs are of particular interest due to their distinct mental health and psychosocial support needs and are therefore the focus of this paper [8, 9].

NDDs are characterised by a complex interplay of genetic and environmental factors affecting cognition and behaviour [10]. This complexity has led to a growing trend of conceptualising neurodevelopmental conditions along a spectrum, emphasising overlapping behavioural phenotypes [11] such as ID, ADHD and autism [12]. These conditions are prevalent worldwide, and idiopathic ID, ADHD and cerebral palsy are among the three most common conditions contributing to developmental disabilities in children under 15 years of age [6, 13].

Children and adolescents with NDDs in LMICs often face barriers to inclusion, including stigma, negative attitudes and beliefs, which place them at heightened risk of neglect and violence [14]. They experience limited access to quality education [14] and have worse health and well‐being outcomes compared to their peers without disabilities [4, 14]. Children and adolescents with NDDs also have an increased risk of mental health problems [15], with challenging behaviours frequently co‐occurring as comorbid conditions [16]. The prevalence of internalising (e.g., anxiety and depression) and externalising (e.g., aggression and non‐compliance) behavioural problems in children with NDDs ranges between 40% and 64%, two to four times higher than the general population [17]. Critically, environmental factors such as parental mental health, socioeconomic status and peer support can influence the negative trajectory of mental health problems [8]. Effective Mental Health and Psychosocial Support (MHPSS) interventions must, therefore, address not only the child but also their broader support systems, including parents, teachers and community stakeholders [9].

School‐based MHPSS interventions offer a solution to the challenge of providing accessible support by enhancing parents' mental health, providing early interventions and addressing mental health problems for children with NDDs [6]. Particularly in LMICs, schools serve as convenient, feasible and cost‐effective settings to deliver children and adolescents MHPSS [18]. Caregivers of children with NDDs also identify schools as critical settings for ‘symptom recognition and subsequent treatment, respite, and support' [19].

However, despite the growing body of literature on inclusive education and mental health interventions in schools, there is limited evidence of their applicability to children and adolescents with developmental disabilities in LMICs [18]. Available systematic reviews focus on specific diagnostic categories (e.g., autism or ADHD) or are limited to high‐income contexts [20]. This gap underscores the pressing need to explore how school‐based interventions in resource‐limited settings can support children and adolescents with NDDs effectively. This study, therefore, aims to identify and evaluate the characteristics and reported effects of school‐based interventions that address mental health and psychosocial support for children and adolescents with NDDs, as well as their parents, teachers and peers in LMICs.

## 2 METHODS

### 2.1 Search strategy and selection criteria

This systematic review was conducted following the preferred reporting Items for systematic reviews and meta‐analysis (PRISMA) 2020 guidelines [21]. The protocol was registered on international prospective register of systematic reviews (CRD42024574717). We searched five databases in October 2024: MEDLINE, Excerpta Medica database Educational Resources Information Center, Global Health and PsycINFO. The search was limited to studies published in English, Spanish and Portuguese since 2008, the year the United Nations Convention on the Rights of Persons with Disabilities (UNCRPD) came into effect [22]. Grey literature databases were excluded.

Our search strategies used Boolean, truncation and proximity operators adapted for each database. The terms used combined keywords related to ‘children and adolescents with neurodevelopmental disabilities,’ ‘school‐based interventions,’ and ‘low‐ and middle‐income countries’ as defined by the World Bank as of June 2024 [23]. An example search strategy is provided in Appendix A.

Studies were eligible if they included children and adolescents attending school grades ranging from primary to secondary or high school levels, as defined by national education systems. To account for differences in school entry age and grade structure across countries, all children under the age of 18 were included. Study settings included both special education and mainstream schools. Preschool and university settings were excluded to ensure a more consistent focus on school‐aged populations.

In addition, eligible studies that reported outcomes related to children and adolescents with NDDs, their parents, peers or teachers were included. Eligible NDD diagnoses included ADHD, autism spectrum disorder, cerebral palsy, epilepsy, foetal alcohol syndrome and idiopathic developmental or intellectual disabilities. Interventions were required to be school‐based and aimed at improving mental health outcomes or providing psychosocial support for children and adolescents with NDDs and/or their parents, teachers or peers.

There was no limitation to eligibility based on primary outcome, which could include depression, anxiety, social skills, participation, behavioural changes or attitudinal shifts among the target groups or other outcomes related to mental health. Quantitative study designs, including randomised controlled trials (RCTs), quasi‐experimental studies, controlled and uncontrolled pre‐post studies and qualitative evaluations of intervention feasibility and acceptability, were included. We excluded studies without disaggregated data for NDD subgroups, case studies and general literature reviews without intervention.

### 2.2 Data analysis

Two researchers (MJADL and RTdS) screened articles independently by title and abstract. The full text was retrieved if the article was relevant to at least one of the reviewers. Citations were imported into Rayyan for file management and decision recording, with duplicates removed. A manual search of references from relevant systematic reviews, evidence and gap maps and citations from included papers was performed. Full‐text screening was managed in EndNote. Both researchers reviewed the full texts independently. Discrepancies were discussed, and consensus was reached. MJADL performed data extraction using an extraction table in Excel. Extracted data included study design, participant characteristics, intervention details (method of delivery, number of sessions, duration, content and delivery agent), outcome measures, findings related to implementation and key results. All extracted data were checked for accuracy by a second reviewer (RTdS).

The quality of the included studies was assessed independently by two reviewers (MJADL and RTdS) using the Joanna Briggs Institute (JBI) Critical Appraisal Tools [24]. Checklists were applied based on the study design, including RCTs, quasi‐experimental studies and qualitative studies. The risk of bias was categorised as ‘low,’ ‘moderate,’ or ‘high.’ In qualitative studies, risk of bias was determined by summing ‘yes’ scores: in qualitative studies 0–4 indicated high risk, 5–7 indicated moderate risk and 8–10 indicated low risk as reported in other reviews [25]. For RCTs and quasi‐experimental studies, a low risk of bias was indicated by 70% or more ‘yes’ responses, moderate risk by 50%–70% and high risk by below 50% as reported in other reviews [26]. Statistical conclusion validity was excluded from the overall assessment [27].

We undertook a narrative synthesis of study results based on their design and intervention strategy. Key characteristics, such as geographical distribution, target populations and the specific NDDs addressed, were summarised. Outcomes were then further categorised according to the previous Evidence and Gap Map on MHPSS interventions for children/adolescents [18].

We classified intervention content using an adapted Community‐Based Rehabilitation (CBR) framework for inclusive interventions for children with disabilities in LMICs [28] in the following domains:

1. Health: access to specialised services for rehabilitation.
2. Education: lifelong learning interventions in social and independent living skills and strengthening the education system through training of teachers and school staff.
3. Awareness and non‐discrimination: media or information campaigns.
4. Protection: interventions to prevent violence/abuse.
5. Adequate standard of living: skills training for work and independence.
6. Family and community life: community support for individual and family relationships and community social activities.
7. Empowerment: peer support groups, advocacy and community mobilisation.

## 3 RESULTS

**FIGURE 1:**
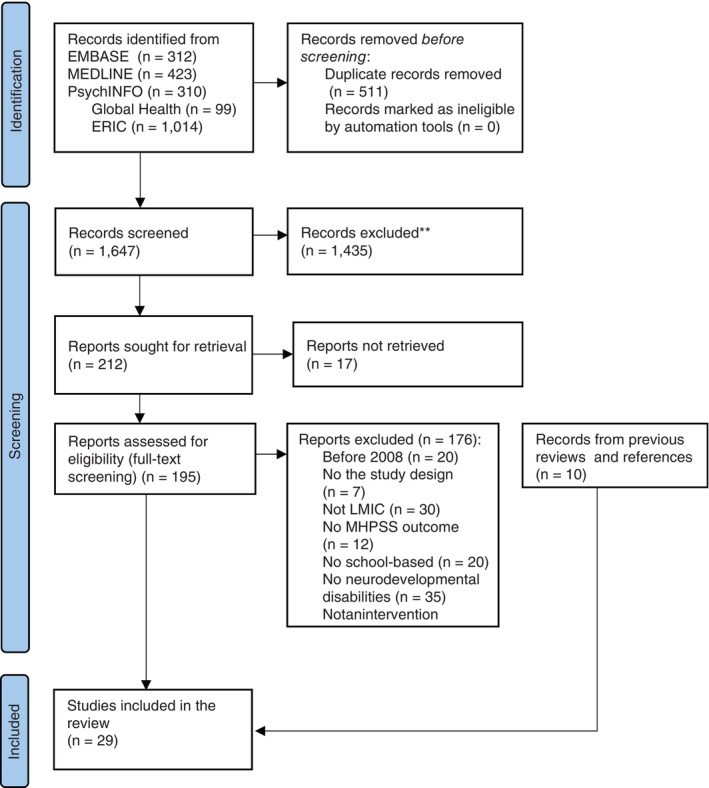
PRISMA flow chart.

The systematic search identified 2158 studies. After removing 511 duplicates, 1647 studies underwent title and abstract screening. Of these, 1435 studies were excluded for not meeting eligibility criteria, leaving 212 records for full‐text retrieval. Seventeen records were unavailable in full‐text format. Following full‐text screening, 176 studies were excluded for reasons outlined in Figure 1. Thirteen systematic reviews identified through the search were reference screened, resulting in 10 additional studies that met the inclusion criteria. Ultimately, 29 studies were included in this review. The full PRISMA search strategy flow is shown in Figure 1.

### 3.1 Study characteristics

Of the 29 included articles, 19 (65.5%) employed a before‐and‐after comparison design. Seven studies used a controlled pre‐post design [29, 30, 31, 32, 33, 34, 35], while 12 employed a pre‐post design without a control group [36, 37, 38, 39, 40, 41, 42, 43, 44, 45, 46, 47]. The review included nine RCTs (31.0%) [48, 49, 50, 51, 52, 53, 54, 55, 56] and one qualitative study (3.0%) exploring the feasibility and acceptability of an intervention [57].

The geographical distribution covered 13 countries across six World Health Organization (WHO) regions. The African region (*n* = 7) and the Southeast Asian region (*n* = 7) each accounted for 24.0% of the studies. India was the most represented country, with six studies. The remaining studies were distributed as follows: five in the Eastern Mediterranean Region (17.0%), four in the European Region (14.0%), four in the Region of the Americas (14.0%) and two in the Western Pacific Region (7.0%) (Table 1).

**TABLE 1:** Summary of characteristics of included studies (*n* = 29).

Characteristic		*N* (%)
Study design	Pre‐post test quasi‐experimental design	**19 (66.0%)**
	Pre‐post no control group	12 (42.0%)
	Controlled pre‐post studies	7 (24.0%)
	Randomised controlled trial	9 (31.0%)
	Qualitative study	1 (3.0%)
WHO region	African Region	**7 (24.0%)**
	European Region	4 (14.0%)
	Western Pacific Region	2 (7.0%)
	Region of the Americas	4 (14.0%)
	Eastern Mediterranean Region	5 (17.0%)
	South East Asia Region	**7 (24.0%)**
Outcomes^a^	NDD symptom severity	4 (9.3%)
	Anxiety	1 (2.3%)
	Depression	2 (4.5%)
	Hopelessness	1 (2.3%)
	Functioning	2 (4.7%)
	Family functioning	2 (4.7%)
	Social skills	**7 (16.3%)**
	Ability to cope (burn out/stress)	3 (6.9%)
	Quality of life	1 (2.3%)
	Knowledge of condition	4 (9.3%)
	Self‐efficacy	1 (2.3%)
	Knowledge attitudes and practices	5 (11.6%)
	Problem behaviour	4 (9.3%)
	Executive functioning	2 (4.6%)
	Development (language, motor and cognitive)	1 (2.3%)
	Sexual abuse prevention	1 (2.3%)
	Academic performance	1 (2.3%)
	Participation	1 (2.3%)
Intervention strategy^a^	Individual intervention	2 (7.0%)
	Physical activity	2 (7.0%)
	Group intervention	5 (17.2%)
	REBT group therapy	2 (7.0%)
	Psychoeducation group intervention	3 (10.3%)
	Educational workshop	**6 (20.6%)**
	Educational materials	1 (3.4%)
	Peer support	1 (3.4%)
	Multimodal intervention (i.e., individual and group)	**6 (20.6%)**
	No specified	1 (3.4%)
Type of inclusive intervention^a^ (CBR adapted framework)	Education	
	Lifelong learning	**11 (33.3%)**
	Educational system strengthening	10 (30.3%)
	Family and community life	
	Community support services	6 (18.2%)
	Health	
	Access to specialist services	2 (6.1%)
	Awareness and no discrimination	
	Information campaign	2 (6.1%)
	Protection	
	Violence and abuse prevention	1 (3.0%)
	Empowerment	
	Advocacy and community mobilisation	1 (3.0%)
Target group	Children with disabilities	**14 (48.0%)**
	Peers without disabilities	2 (7.0%)
	Parents/caregivers	5 (17.0%)
	Teachers	7 (24.0%)
	Parents and parents	1 (4.0%)
Educational setting	Mainstream school/inclusive school	13 (45.0%)
	Special education school	**15 (52.0%)**
	Not reported	1 (3.0%)
Target impairment^a^	Autism	8 (23.0%)
	Intellectual disability	**12 (34.0%)**
	Epilepsy	6 (17.0%)
	Attention deficit hyperactivity disorder	8 (23.0%)
	Cerebral palsy	1 (3.0%)
Risk of bias	High	5 (17.0%)
	Moderate	**21 (73.0%)**
	Low	3 (10.0%)

*Note*: Bold letters highlight the most frequent characteristics.

Abbreviations: CBR, Community‐Based Rehabilitation; NDD, neurodevelopmental disorder; REBT, rational emotional behavioural therapy.

^a^ Means that some interventions targeted more than one group, strategy or outcome.

### 3.2 Target population characteristics

The target population and characteristics varied considerably between studies (Table 1). The largest number of studies focused on children and adolescents with neurodevelopmental disabilities (*n* = 14, 48.0%), followed by studies on teachers (*n* = 7, 24.0%), parents or caregivers (*n* = 5, 17.0%) and peers without disabilities (*n* = 2, 7.0%). One study focused on both children and parents, measuring outcomes for both groups [55].

Children and adolescents targeted by the studies ranged in age from 5 to 21 years. Sample sizes ranged from 15 [37] to 230 [41] participants. For caregiver‐focused interventions, participants' ages ranged from 36 to 49 years. Two studies specifically targeted mothers [35, 50], three included both mothers and fathers [39, 57] and one involved caregivers in general [38]. Studies that targeted peers without disabilities focused on adolescents aged 14–20 years [43, 44] (Table 2).

**TABLE 2:** Design of included studies.

First author (year)	Country	Study design	Length of follow‐up after intervention	Intervention target group/condition targeted (age)	Sample size (sex)	Outcome targeted	Method of assessment
Adeniyi (2016) [36]	Nigeria	Pre‐post QED with a single group	N/F	Children‐adolescents/ID (12–19 years)	30 (*m* = 16, *f* = 14)	Social skills	Parent/teacher reported scale
Adibsereshki (2015) [29]	Iran	Pre‐post QED with control group	N/F	Children/autism (intervention, mean = 9.58, control mean = 9.75)	24 (*m* = 12, *f* = 12)	Social skills	Parent/teacher reported scale
Aguiar (2014) [47]	Brazil	Pre‐post QED with a single group	N/F	Teachers/ADHD	37 (*f* = 37)	Knowledge about ADHD symptoms and management	Self‐reported questionnaire
Alavi (2013) [32]	Iran	Pre‐post QED with control group	N/F	Children/ID (8–10 years)	40 (*m* = 40)	Aggressive behaviour	Self‐reported scale
Cenk (2016) [38]	Turkey	Pre‐post QED with a single group	N/F	Caregivers/ID (males mean = 44.75, females mean = 39.82)	104 caregivers (mothers: 83, fathers: 16, other: 5)	Knowledge, family burden and hopelessness	Self‐reported scale
Eze (2015) [40]	Nigeria	Pre‐post QED with a single group	3 months	Teachers/epilepsy (18–56 years)	226 (*m* = 71, *f* = 155)	Knowledge, attitudes and first aid epilepsy management skills	Self‐reported questionnaire
Goel (2014) [42]	India	Pre‐post QED with a single group	3 months	Teachers/epilepsy (mean = 40.8)	85 (*m* = 16, *f* = 69)	Knowledge, attitudes and first aid epilepsy management	Self‐ administered questionnaire
Haack (2021) [52]	Mexico	RCT: pilot	N/F	Children/ADHD (mean intervention = 7.3, mean control = 7.6)	58 (*m* = 42, *f* = 16)	ADHD/ODD symptoms functional impairment	Parent/teacher reported scale
Haack (2024) [53]	Mexico	RCT	N/F	Children/ADHD (6–12 years)	57 (*m* = 42, *f* = 15)	ADHD/ODD symptoms functional impairment	Parent/teacher reported scale
Ireri (2019) [30]	Kenya	Pre‐post QED with control group	N/F	Children‐adolescents autism (5–21 years)	40 (*m* = 27, *f* = 13)	Anxiety Social deficits	Parent reported scale
Kalgotra (2019) [31]	India	Pre‐post QED with control group	N/F	Children/ID (6–17 years)	70 (*m* = 42, *f* = 28)	Social skills	Semi‐structured interview with teachers/parents and children's observation
Kok (2015) [39]	Turkey	Pre‐post QED with a single group	1 month	Parents/ID (40–49 years)	84 (*m* = 42, *f* = 42)	Self‐efficacy Knowledge of sexual development	Questionnaire administered by researcher
Kolar (2016) [44]	India	Pre‐post QED with a single group	N/F	Peers/epilepsy (mean = 14.55)	70 (*m* = 38, *f* = 32)	Knowledge, attitudes and practices	Questionnaire administered by researcher on an interview
Kurani (2009) [34]	India	Pre‐post QED with control group	N/F	Children/ID, autism, epilepsy, CP (4–12 years)	22 (*m* = 18, *f* = 4)	Motor, cognitive, language, social and self‐help development	Scale completed after children's observation and interview with parents.
Lan (2020) [54]	China	RCT	3 months	Children/ADHD (9–12 years)	81 (*m* = 52, *f* = 29)	Primary outcome: peer relationship	Parent/teacher reported scale
						Secondary outcomes: executive function, social skills, ADHD symptoms	Battery of computerised psychometric task, face to face psychometric test and parent/teacher reported scale
Mero (2024) [51]	Ecuador	RCT	N/F	Children/ID (10–14 years)	30 (*m* = 16, *f* = 14)	Attention and inhibitory control	Computer‐based task
Naheed (2022) [35]	Bangladesh	Pre‐post QED with control group: feasibility trial	N/F	Mothers/autism (mean = 41)	188 (*f* = 188)	Depression Quality of life	Scales administered by psychologist to mothers
Nalugya (2023) [57]	Uganda	Qualitative: acceptability and feasibility study	N/F	Parents/ID, autism (median = 36)	64 (*m* = 7, *f* = 57)	Implementation outcomes and participation of children with disabilities	Interviews, focus groups, consultative meeting and evaluation and feedback workshops with parents
Obiweluozo (2021) [56]	Nigeria	RCT	3, 6 months	Teachers/autism, ADHD, ID (mean intervention = 31, mean control = 33)	87 (*m* = 29, *f* = 58)	Stress	Self‐reported scale
Ozcan (2013) [45]	Turkey	Pre‐post QED with a single group	N/F	Children/ADHD (6 to 11 years)	33 (*m* = 30, *f* = 3)	Behavioural and emotional problems	Parent/teacher reported scale
Pasha (2010) [33]	Iran	Pre‐post QED with control group	N/F	Children/ID (not reported)	70 (*m* = 42, *f* = 28)	Social skills Behavioural disorders	Interview with children or parent, children's observation and parent/teacher reported scale
Shanker (2023) [48]	India	RCT	N/F	Children‐adolescents autism (5–15 years)	43 (NR)	Social responsiveness Problem behaviour	Teacher reported scale
Shen (2021) [55]	China	RCT	4 and 10 months	Children/ADHD (6–11 years) Parents (mean fathers = 36, mean mothers = 34)	204 (*m* = 175, *f* = 29) Parents (not reported)	ADHD symptoms Academic performance Parents stress	Scales completed by parents and teachers
Sulena (2023) [41]	India	Pre‐post test with single group	N/F	Teachers/epilepsy (mean = 43)	230 (*m* = 109, *f* = 121)	Knowledge, attitudes and practices	Self‐reported questionnaire
Syed (2010) [46]	Pakistan	Pre‐post QED with a single group	6 months	Teachers/ADHD (mean = 26 years)	49 (*f* = 49)	Knowledge of signs and symptoms	Self‐reported scale
Tekle‐Haimanot (2016) [43]	Ethiopia	Pre‐post test with single group	N/F	Peers/epilepsy (16–20 years)	226 (*m* = 106, *f* = 120)	Knowledge, attitudes and practices	Self‐reported scale
Uzodinma (2022) [49]	Nigeria	RCT	6 months	Teachers/autism (not reported)	86 (*m* = 29, *f* = 59)	Burn out	Self‐reported scale
Warraitch (2021) [37]	Pakistan	Pre‐post QED with a single group: feasibility trial	N/F	Children/ID (10–15 years)	15 (*f* = 15)	Sexual abuse prevention	Questionnaire administered by researcher
Yildirim (2013) [50]	Turkey	RCT	N/F	Mothers/ID (not reported)	75 (*f* = 75)	Risk of depression Family function	Scales administered by researcher to the mother on an interview

Abbreviations: ADHD, attention deficit hyperactivity disorder; CP, cerebral palsy; *f*, female; ID, intellectual disability; *M*, mean, *m*, male; N/F, no follow‐up; ODD, oppositional defiant disorder; QED, quasi‐experimental design; RCT, randomised controlled trial.

The studies targeted ID, autism, ADHD and epilepsy, with one also including cerebral palsy, though not as the primary focus [34]. Three studies addressed multiple impairments [34, 56, 57]. ID was the most studied (*n* = 12, 34.0%), followed by autism (*n* = 8, 23.0%), ADHD (*n* = 8, 23.0%) and epilepsy (*n* = 6, 17.0%). No studies focused on foetal alcohol syndrome (Table 2).

### 3.3 Outcomes

Social skills improvement was the most common targeted effect (*n* = 7, 16.3%) [29, 30, 31, 33, 36, 54] (Table 2). Most interventions effectively enhanced social skills or reduced deficits in children with autism, intellectual disabilities and ADHD. Table 3 describes the reported effects of the studies. Different strategies were used in interventions that aimed to improve social skills, leading to varying effects. Multimodal interventions resulted in broad improvements, though Kalgotra et al. [31] found greater skill enhancement in children with mild intellectual disabilities than in those with moderate disabilities. Group interventions also improved social skills in children with ADHD and intellectual disabilities [36, 54], while individualised Theory of Mind training improved social skills in children with autism [29]. A computer‐based intervention targeting executive functioning [54] enhanced peer interactions and reduced peer‐related issues, with effects persisting at 3 months. A yoga intervention [48] improved social communication in children with autism but had no significant impact on overall social skills.

**TABLE 3:** Intervention characteristics and study findings.

First author (year)	Educational Setting	Intervention delivery methods	Duration of intervention	Delivery agent	Intervention content	Key findings	Risk of bias
Autism							
Adibsereshki (2015) [29]	NR	Individual	5 weeks	Researcher and special education students	Sessions focused on ToM, including emotions, desires, beliefs and situational understanding	Teachers reported improved social skills in students after the intervention (average score on intervention group 29.08 vs. control group 24.08, *p* = 0.001)	Low
Shanker (2023) [48]	Special education school	Physical activity group intervention	12 weeks	Yoga teacher	Integrated Yoga Therapy, including chanting, breathing exercises, yoga poses and relaxation	Moderate effect on social communication (Cohen's *d* = 0.724, *p* = 0.021), no effect on overall social responsiveness. Problem behaviours decreased with moderate effects on irritability (Cohen's *d* = 0.637, *p* = 0.041) and large effect on social withdrawal (Cohen's *d* = 0.909, *p* = 0.047)	Moderate
Uzodinma (2022) [49]	Special education school and mainstream school	REBT manualised group therapy	12 weeks	Researcher and research assistants	REBT covering problem‐solving, cognitive restructuring and healthy coping, with practical exercises	Decrease in self‐reported burn‐out, sustained at 6‐month follow‐up (*η* ^2^ = 0.62, *p* = 0.001). High competence and adherence in delivering intervention	Moderate
Naheed (2022) [35]	Special education school	Individual intervention, group workshops and home visits	6 months	Psychologist	Psychoeducation, cognitive restructuring and skill development for community engagement, complemented by mental health awareness workshops and home visits	Decrease in depression in the intervention group (average score from 79.5 to 60, *p* = 0.004). Improvement in quality of life (from 70.3 to 80.2, *p* = 0.001), with significant changes in pain/discomfort and anxiety/depression domains. Feasibility: 95% of mothers satisfied, 85% attended sessions, 75% completed exit interview	Moderate
Ireri (2019) [30]	Special education school	CBT individual and group Intervention, family and school involvement	6 months	Psychologists and teachers	Multimodal CBT‐based intervention, including psychoeducation, graded exposure, group social skills practice and family/school involvement	Decrease in parent reported social deficits (*η* ^2^ = 0.093, *p* = 0.023)	Low
Intellectual disability							
Adeniyi (2016) [36]	Special education school	Classroom based group intervention	8 weeks	Teachers	Curriculum for communication and social interaction skills, using narrative and role‐play methods	Parents or teachers reported improvement in social skills in students after intervention (average score: 126.6 vs. 136.0, *p* = 0.001). Fidelity: the content was covered by teachers between 65% and 85%	Moderate
Yildirim (2013) [50]	Special Education School	Psychoeducational group intervention	4 weeks	Nurses	Psychosocial education covering care, communication, problem‐solving and available support institutions	Decrease in depression risk on intervention group in comparison to control (average score 12.13 vs. 18.80, *p* = 0.001) and improvement on family function perception (average score 1.71 vs. 2.74, *p* = 0.001)	High
Mero (2024) [51]	Special education school	Physical activity group intervention	6 weeks	Researcher and physical education teacher	Enriched physical education focusing on attention and inhibitory control through progressively challenging games	Moderate effect in reaction time (*β* = −0.247, *p* = <0.001), large effect on accuracy (*β* = 0.639, *p* < 0.05) indicating improvement on focused attention. No effect on inhibitory control	Moderate
Warraitch (2021) [37]	Special education school	Group intervention	5 weeks	Researcher	Child sexual abuse prevention focusing on body ownership, private parts, appropriate/inappropriate behaviour, refusal and reporting, using role‐play and feedback	Increase on students' knowledge and skills on sexual abuse prevention (from 9.47 vs. 48.40, *p* = 0.001) Acceptability: 93.7% of students rated the intervention acceptable. Feasibility: 100% of students rated the intervention as feasible	Moderate
Kalgotra (2019) [31]	Special education school	Individual and group intervention	32 weeks	Teachers	Social skills training on interaction, friendship, cleanliness, safety, aggression control, using; mix of verbal instruction, modelling, role‐play and rewards, based on ABA strategies	Increase on social skills on the group of children with mild intellectual disability in comparison to control (average score 67.33 vs. 63.27, *p* = 0.001) and in the group with moderate intellectual disability (average score 46.59 vs. 44.81, *p* = 0.001) with a bigger effect size on the mild intellectual disability group (ES = 0.885)	Moderate
Cenk (2016) [38]	Special education school	Psychoeducation group intervention	3 weeks	Researcher	Semi‐structured education on disability, care, communication, socialisation and rights, delivered via lectures and active learning	Increase on the knowledge of families (average score from 15.91 to 19.64, *p* = 0.001), decrease in the hopelessness scores (from 9.75 to 6.25, *p* = 0.001). Increase in the perceived emotional burden (from 30.48 to 35.54, *p* = 0.001) and time required to care (from 22.60 to 24.67, *p* = 0.044).	Moderate
Alavi (2013) [32]	Special education school	Group intervention	10 sessions	Researcher	Social skills programme on personal, interpersonal and environmental‐related skills, using brainstorming, lectures and practice	Decrease in the self‐reported aggressive behaviour in the intervention group (average score 69.70 vs. 96.30, *p* = 0.001)	High
Pasha (2010) [33]	Special education school	NR	12 weeks	NR	Social skills training covering self‐assistance, emotional regulation and general social skills	Parents and teachers reported improvement in social skills (average score from 74.31 to 75.37, *p* = 0.0001) and decrease in behavioural disorders (average score from 47.86 to 42.71, *p* = 0.0001)	High
Kok (2015) [39]	Special education school	Psychoeducation group intervention	6 weeks	Nurses	Reproductive health education, including hygiene, masturbation control and protection from sexual abuse	Increase in perceived self‐efficacy in mothers (average score from 28.47 to 34.64, *p* = 0.001) and fathers (average score from 26.64 to 33.90). Increase on reproductive health knowledge of mothers (score from 17.47 to 18.59, *p* = 0.001) and fathers (score from 17.08 to 18.26, *p* = 0.007) sustained at 1 month follow‐up. Acceptability: 64.3% of mothers and 69% of fathers rated the intervention as necessary	High
Epilepsy							
Eze (2015) [40]	Mainstream school	Educational workshop	1 time	Researcher	Lecture‐based intervention on epilepsy's biological, psychosocial aspects, risks, triggers and first aid management	After 3 months follow‐up, the proportion of teachers classified with good knowledge increased 29.6% (*p* = <0.0001), the participants classified with positive attitude increased 16.4% (*p* = <0.0001) and the ones classified as good first as management skills increased 25% (*p* = <0.0001)	Moderate
Sulena (2023) [41]	Mainstream school	Educational workshop	1 time	NR	Educational workshop on epilepsy types, special needs and first aid, using multimedia and role‐playing	Knowledge of epilepsy, seizure first aid and positive attitudes towards participation (*p* = 0.001). Negative effects: teachers would allow risky outdoor activities and reject giving extra attention in class. Acceptability: 96% of teachers found the workshop beneficial	Moderate
Goel (2014) [42]	Mainstream school	Educational workshop	Not reported	Researcher	Workshop on epilepsy basics, triggers and first aid management, using manual, presentations and videos	After 3 months follow‐up knowledge, attitudes related to epilepsy, first aid management on hypothetical situations (*p* = 0.001) and attitudes towards marriage (*p* = 0.02)	Moderate
Tekle‐Haimanot (2016) [43]	Mainstream school	Comic book	Students read the comic book one time	NR	Comic book‐based education on epilepsy, focusing on diagnosis, first aid and social integration	Knowledge of epilepsy causes, diagnosis, seizure first aid and attitudes towards marriage (*p* = <0.001). Feedback: 90% of professionals supported distributing the comic	Moderate
Kolar (2016) [44]	Mainstream school	Educational workshop	3 days	NR	Workshop on epilepsy awareness and first aid, using posters, brochures, role plays and group activities.	Improved knowledge (from 3.47 to 7.60, *p* = 0.001), attitudes (from 0.64 to 7.59, *p* = 0.001) and practices (from 1.36 to 7.67, *p* = 0.001)	Moderate
ADHD							
Aguiar (2014) [47]	Mainstream school	Educational workshop	1 day	Researcher	Package of units of learning on ADHD, included: symptoms presentation at school and aetiology, management strategies of ADHD in school. The intervention included a summary manual	Teachers' knowledge of ADHD increased from 14.94 to 17.36 (*p* < 0.001, large effect size *η* ^2^ = 0.57).	Moderate
Ozcan (2013) [45]	Mainstream school	Group intervention	14 weeks	Researcher	Interpersonal cognitive problem‐solving with social skills training using games, stories and role‐playing	Reduction in problem behaviours (attention, hyperactivity‐impulsivity, oppositional defiant) reported by parents and teachers (*p* < 0.001). Reduction in internalising and externalising behaviours (from 84.51 to 62.61, *p* < 0.001). Improvement in competence behaviours (from 12.93 to 13.88, *p* = 0.031)	Moderate
Haack (2021) [52]	Mainstream school	Group intervention for children. Psychoeducation parents group Orientation for teachers	6 weeks	School mental health professional	Collaborative Life Skills programme covering organisation, social skills for children, behaviour management for parents and use of school‐home daily report card for teachers	Reduction on ADHD symptom severity in the parents report (ES = 0.89) and teachers report (ES = 1.04), reduction of the ODD symptom severity in teachers report (ES = 0.78) and in functional impairment reported by parents (ES = 0.89) and teachers (ES = 1.02). Acceptability: 98% of parents found the intervention appropriate Fidelity: 97% of elements covered on parents sessions	Moderate
Haack (2024) [53]	Mainstream school	Group intervention for children (hybrid). Psychoeducation parents group (remote) Orientation for teachers (remote)	6 weeks	School mental health professional	Similar to Haack (2021) but with hybrid delivery and electronic tools for communication between teachers and parents	Reduction of ADHD symptom severity in parents report (ES = 0.48) and teachers report (ES = 0.97). Reduction on ODD symptoms (ES = 0.19) and functional impairment (ES = 0.62) in parents report. Feasibility: 83% students 72% parents attendance Acceptability: 100% of parents and 91% teachers satisfied.	Moderate
Syed (2010) [46]	Mainstream school	Educational workshop	5 days	Researcher and clinical psychologist	Educational workshop on ADHD symptoms, assessment and classroom management, using interactive videos and printed material	After 6 months follow‐up, increase on teachers knowledge on ADHD symptoms (average score from 10.7 to 11.6, *p* = <0.005)	Moderate
Lan (2020) [54]	Mainstream school	Intervention 1: Group computer‐based executive function training (GEFT)	12 weeks	Researcher	GEFT: computer task and group games focused on inhibition, working memory and flexibility	Improvement in peer interaction in GEFT (from 29.78 to 40.21, *p* = 0.05) and SST group (from 31.35 to 39.78, *p* = 0.05). Decrease in problems with peers in GEFT (from 31.76 to 24.21, *p* = 0.05) and SST (from 29.78 to 24.18, *p* = 0.05). Reduction in ADHD symptom, on the GEFT group with moderate effect on inattention (*η* ^2^ = 0.138) and large effect on hyperactivity/impulsivity (*η* ^2^ = 0.14)	Moderate
		Intervention 2: Group social skill training (SST)			SST: focused on social behaviours and emotional regulation, using modelling, roleplaying and reinforcement		
Shen (2021) [55]	Mainstream school	Psychoeducation for parents and teachers Counselling sessions for families	16 weeks	Paediatricians	Training on ADHD knowledge, stress management and communication, with separate sessions for parents and teachers	After 10 months follow‐up, improvements on intervention group in comparison to control on ADHD symptoms (average score 23.98 vs. 30.07, *p* = 0.005), academic performance (47.55 vs. 45.42, *p* = 0.001) and reduction on parent stress (71.99 vs. 75.00, *p* = 0.042). Acceptability: 64.8% rated the treatment as helpful	Moderate
Multiple impairments							
Kurani (2009) [34]	Special education school	Individual parent training as co‐therapist	5 days	Special education teacher	Learning readiness programme focusing on attending skills, social and motor skills, communication and self‐help, with parents as co‐therapists	Improvements on motor development, language development, socialisation (*p* = 0.001) and self‐help behaviours (*p* = <0.001) in children of parents with high parental involvement. No significant changes in the group of low parental involvement	High
Nalugya (2023) [57]	Mainstream school	Peer to peer support intervention	2 years	Disability inclusion experts and peer parents	Peer‐to‐peer support intervention promoting inclusion, belonging and cultural values through training and community activities	Parents found the intervention inspiring, acceptable, culturally appropriate and supportive, build in the values of their cultural tradition. Enhanced a sense of togetherness and belonging and develop more positive attitudes towards children with disabilities and disability inclusion in home, school and communities	Low
Obiweluozo (2021) [56]	Special education school	REBT manualised group intervention (hybrid)	12 weeks	Researcher	REBT focusing on cognitive restructuring and behaviour management, delivered in hybrid format	After 6 months follow‐up, reduction on teacher's perceived stress, large effect size (*η* ^2^ = 0.7, *p* = 0.0001). Acceptability: 86.2% teachers satisfied	Moderate

Abbreviations: ABA, Applied Behaviour Analysis; ADHD, attention deficit hyperactivity disorder; CBT, Cognitive Behavioural Therapy; ES, effect size; *f* = female; *M*, mean, *m*, male; NR, not reported; ODD, oppositional defiant disorder; REBT, Rational Emotive Behavioural Therapy; ToM, Theory of Mind; *η* ^2^ partial Eta squared (effect size), β‐standardised regression coefficient.

Interventions to improve teachers' and peers' knowledge, attitudes and practices regarding epilepsy [40, 41, 42, 43, 44] increased knowledge about the causes and diagnosis of epilepsy, fostered positive attitudes towards children's participation in the community and improved first‐aid skills in hypothetical situations. However, one study [41] reported a negative shift in teachers' attitudes towards allocating additional attention in the classroom to children with epilepsy.

Interventions that measured changes in the severity of ADHD symptoms [52, 53, 54, 55] showed mixed results. Multimodal and computer‐based interventions were associated with a reduction in reported symptoms such as inattention and hyperactivity/impulsivity. In contrast, social skills group intervention did not result in a reduction of symptoms.

Problematic behaviour in children with autism, ID and ADHD was reduced after group interventions focused on social skills [32] and problem‐solving [45]. Furthermore, implementing a yoga intervention [48] demonstrated a reduction in irritability and social withdrawal, yet no discernible impact on global measurements of problematic behaviour. However, there was no follow‐up reported in these interventions.

Interventions that focused on teachers' and parents' ability to cope showed a reduction in teacher burn‐out [49] that was maintained for 6 months, a decrease in reported parental stress [55] that was maintained for 10 months and a reduction in teachers' self‐reported stress [56] after 6 months of follow‐up.

In four studies, there was an increase in parents' knowledge of reproductive health [39], rights and care [38] of children and adolescents with ID and in teachers' knowledge of ADHD signs and management [46, 47].

Two interventions [52, 53] for children with ADHD reported a reduction in problems related to interaction with the family and peers and an improvement in academic progress. Both interventions were multimodal; one was conducted in person [52], while the other was in a hybrid format [53]. However, no follow‐up was conducted.

The effects of interventions for caregivers of children with ID on family function obtained mixed results. While a psychoeducation group for mothers focused on problem‐solving and care enhanced the perception of family functioning [50], another psychoeducation group led to an increased perception of emotional burden and time required for caring [38].

Interventions related to executive functioning did not show improvement in all the targeted cognitive functions. The physical education intervention [51] and computer‐based task [54] enhanced the performance of children with ID and ADHD in reaction time, focused attention and working memory. The effects of the computer‐based intervention were sustained at a 3‐month follow‐up.

Psychoeducational groups [38, 50] and multimodal interventions, including individual and group intervention as well as home visits [35] reduced depression scores in mothers, improved quality of life [35] and reduced hopelessness [38].

A multimodal intervention involving cognitive behavioural therapy [30] did not reduce anxiety levels in children with autism. An intervention that trained parents to act as co‐therapists for their children [34] improved motor, language and social development in children, but only when parents were highly involved with their children. A group intervention for girls with ID [37] increased their knowledge and skills in identifying risk situations and preventing sexual abuse. Academic performance in children with ADHD improved and was sustained for 10 months, following a multimodal intervention involving psychoeducation for parents, family counselling and school involvement [55].

Finally, a peer‐to‐peer intervention [57] increased the participation of children with disabilities as parents became involved in their child's learning process, encouraged participation in household activities and developed more positive attitudes about their children's abilities.

### 3.4 Intervention characteristics

Many interventions were researcher‐led (*n* = 13, 45.0%), with 10 conducted exclusively by researchers [32, 37, 38, 40, 42, 45, 47, 49, 54, 56]. Two were co‐delivered with teachers, [29, 51] and one involved a psychologist [46]. Teachers implemented seven interventions, either independently [29, 31, 34, 36, 48, 51] or with psychologists [30]. School mental health professionals led five [30, 35, 46, 52, 53], health professionals delivered three [39, 50, 55] and one was facilitated by peer parents and disability inclusion experts [57]. Four studies did not specify delivery agents [33, 41, 43, 44].

The most common educational setting was special education schools (*n* = 15, 52.0%). Thirteen studies (45.0%) were conducted in mainstream schools, involving educational workshops for teachers and peers related to epilepsy and ADHD and interventions targeted to children and adolescents with ADHD. The only intervention conducted in a mainstream school that involved parents of participants with intellectual disabilities and autism was Nalugya et al. [57]. Programme durations ranged from single‐session workshops to 32 weeks.

The studies included a variety of intervention strategies. The most common intervention strategies were multimodal approaches and educational workshops, each used in 20.6% (*n* = 6). Multimodal interventions combined individual and group components [30, 31, 35, 52, 53, 55], targeting children and adolescents with autism, ID, ADHD and their parents. Many also incorporated school‐family involvement, including psychoeducation for teachers and guidance on classroom strategies (*n* = 4) [30, 52, 53, 55]. Educational workshops focused on teacher training for epilepsy and ADHD and peer training for epilepsy [40, 41, 42, 44, 46, 47].

Five studies were group‐based interventions [32, 36, 37, 45, 54] targeting children and adolescents with ID (*n* = 3) and ADHD (*n* = 2). Three studies [38, 39, 50] employed psychoeducation groups, which were designed for caregivers of children with intellectual disabilities. Individual interventions (*n* = 2) [29, 34] targeted children with autism, epilepsy and ID.

Physical activity‐based interventions were reported in two studies [48, 51]. These included a yoga therapy programme for children with autism and an enriched physical education programme for children with intellectual disabilities.

Two studies [49, 56] employed manualised Rational Emotive Behavioural group therapy for teachers of children with neurodevelopmental disorders, with one delivered in a hybrid format.

Other less frequently reported intervention strategies included a computer‐based intervention paired with group games [54], the distribution of an educational comic book on epilepsy [43] and a peer‐to‐peer support intervention [57] for parents of children with disabilities.

### 3.5 Intervention content

Eleven studies focused on lifelong learning [29, 30, 31, 32, 33, 45, 48, 52, 53, 54] according to intervention content based on the adapted CBR framework [28] (Table 4). These studies covered emotional regulation, relaxation techniques, social skills, personal hygiene and organisational abilities. Ten studies targeted educational system strengthening, training teachers in problem‐solving, cognitive restructuring and behaviour management and also included ADHD and epilepsy recognition and seizures first aid [40, 41, 42, 46, 47, 49, 52, 53, 55, 56]. Community support interventions (*n* = 6) provided caregiving strategies, community engagement, disability rights awareness, stress management and reproductive health education, including personal hygiene and abuse prevention [34, 35, 38, 39, 50, 55]. Two interventions focused on cognitive rehabilitation, addressing executive functions such as inhibitory control, attention, working memory and cognitive flexibility [51, 54]. Information campaigns (*n* = 2) educated peers on epilepsy, its diagnosis, first aid and social inclusion [43, 44]. One intervention addressed child protection by educating girls with intellectual disabilities on body ownership, appropriate behaviour and abuse prevention [37], while another focused on empowerment, advocacy and community mobilisation by fostering parental peer support networks to promote inclusion and belonging [57].

**TABLE 4:** Community‐Based Rehabilitation adapted framework to analyse the intervention content.

Intervention domain	Education		Family and community life	Health	Awareness and no discrimination	Protection	Empowerment
Intervention type	Lifelong learning	Educational system strengthening	Community support services	Access to specialist services	Information campaign	Violence and abuse prevention	Advocacy and community mobilisation
Adeniyi and Omigbodun [36]	X						
Adibsereshki et al. [29]	X						
Aguiar et al. [47]		X					
Alavi et al. [32]	X						
Cenk et al. [38]			X				
Eze et al. [40]		X					
Goel et al. [42]		X					
Haack et al. [52]	X	X					
Haack et al. [53]	X	X					
Ireri et al. [30]	X						
Kalgotra et al. [31]	X						
Kok and Akyuz [39]			X				
Kolar et al. [44]					X		
Kurani et al. [34]			X				
Lan et al. [54]	X			X			
Mero et al. [51]				X			
Naheed et al. [35]			X				
Nalugya et al. [57]							X
Obiweluozo et al. [56]		X					
Ozcan et al. [45]	X						
Pasha and Gorjian [33]	X						
Shanker and Pradhan [48]	X						
Shen et al. [55]		X	X				
Sulena et al. [41]		X					
Syed et al. [46]		X					
Tekle‐Haimanot et al. [43]					X		
Uzodinma (2022) [49]		X					
Warraitch et al. [37]						X	
Yildirim et al. [50]			X				

### 3.6 Risk of bias

Twenty‐one studies (73.0%) were rated as having a moderate risk of bias, five studies (17.0%) were rated as having a high risk of bias, and three studies had a low risk of bias (10.0%). Potential sources of bias among those rated as moderate to high risk of bias included reliance on single measurements taken pre‐intervention and post‐intervention and absence of effect size calculations. The RCTs lacked blinding for participants or outcome assessors and did not include statistical power calculations.

## 4 DISCUSSION

This review identified 29 studies on school‐based MHPSS interventions for children and adolescents with NDDs, including their parents, teachers and peers in LMICs. The most common strategies were multimodal interventions, comprising individual and group components and educational workshops. Most intervention content focused on lifelong learning and educational system strengthening. For example, a parent and teacher training on ADHD knowledge, stress and management, a child group programme that uses stories and role‐play to promote social skills, and a group yoga intervention for children that included breathing and relaxation to address problem behaviour and social responsiveness demonstrate the diversity of the interventions that can be used to support children and adolescents with NDD. Social skills and changes in knowledge, attitudes and practices were the most frequently targeted primary outcomes. The identified interventions were, by and large, found to be effective in supporting the mental health and well‐being of children and adolescents with NDD. Positive effects included improved peer relationships and increased epilepsy awareness; nevertheless, the heterogeneity of outcome measures affects the ability to compare results across studies and draw consistent generalisable results. In addition, most studies were quasi‐experimental with moderate risk of bias and small samples. These factors further reduce generalisability, as findings may be influenced by individual differences, contextual factors or external events rather than the intervention itself, so these results should be interpreted with caution.

Findings align with research from high‐income settings, where school multimodal interventions effectively address behavioural and social challenges in children with NDDs [17]. However, gaps remain in LMIC‐focused research. Peer‐based interventions, proven to foster inclusion in high‐income countries [58, 59], were sparse and primarily targeted adolescents in this review, despite evidence that early peer interactions enhance acceptance and inclusion [60]. Additionally, in high‐income countries, many school‐based mental health interventions target children with NDDs in mainstream education [17, 61, 62]. Over half (52%) of the included studies were conducted in special education schools, and the studies in mainstream settings primarily targeted ADHD students, likely due to limited access to inclusive education in LMICs [63, 64].

Future research should expand to underrepresented conditions that were less directly targeted in the interventions reviewed, such as epilepsy, cerebral palsy and foetal alcohol syndrome. These groups, which were not included in the current review, also experience heightened risks of mental health issues and warrant greater attention [6, 65, 66, 67, 68]. Future research should also include children with complex needs and comorbidities, reflecting real‐world conditions where the co‐occurrence of multiple conditions is common [69, 70, 71]. Studies may also consider prioritising validated self‐report adapted measures such as picture‐based scales [72] over proxy assessments to reduce response bias and capture children's perspectives. Research is needed on school‐based interventions that address stigma, discrimination and advocacy to promote disability inclusion and empowerment. Additionally, long‐term follow‐up assessments should be incorporated to evaluate the impact of interventions.

### 4.1 Strengths and limitations

This review highlights the potential of school‐based MHPSS interventions in LMICs, addressing systemic barriers to mental health support by involving children, caregivers, teachers and peers. Strengths of the study include a robust systematic review and data extraction process. However, the inclusion of only three languages may have limited the scope of eligible studies. Additionally, the focus on NDDs means that findings may not be generalisable to all children with disabilities, such as sensory impairments (e.g., visual impairment or hearing impairment), who may have different needs and support requirements.

## 5 CONCLUSIONS

Schools inherently bring together children, peers, families and teachers, making them strategic platforms for delivering psychosocial interventions. This review found that most studies focussed on intellectual disabilities, autism and ADHD, with workshops and multimodal approaches being the most common delivery strategy. However, key gaps remain. There is limited integration of community empowerment and child protection components. Expanding interventions into mainstream schools and addressing the barriers to inclusive education in LMICs should be a research priority. Schools offer a promising foundation for MHPSS delivery, but more rigorous and inclusive research is needed to inform effective implementation.

## FUNDING INFORMATION

Tracey Smythe is supported by the Medical Research Council (grant number UKRI165). The funder had no role in the study design, data collection, data analysis, data interpretation or writing of the manuscript.

## APPENDIX A SEARCH TERMS

**Table:**

Category	Search terms
LMICs	developing country/ or low income country/ or middle income country OR ((developing or less* developed or under developed or underdeveloped or middle income or low* income) adj (economy or economies)) OR((developing or less* developed or under developed or underdeveloped or middle income or low* income or underserved or under served or deprived or poor*) adj (countr* or nation? or population? or world) OR (low* adj (gdp or gnp or gross domestic or gross national)) OR(low adj3 middle adj3 countr*) OR (lmic or lmics or third world or lami countr*) OR transitional countr* OR global south OR Africa south of the Sahara OR “Africa South of the Sahara” or sub‐Saharan Africa or subSaharan Africa OR Central Africa OR Eastern Africa OR Southern Africa OR Western Africa OR North Korea OR (North Korea or (Democratic People* Republic adj2 Korea)) OR Haiti OR(Haiti or Hayti) OR Afghanistan OR Nepal OR Syrian Arab Republic OR Yemen OR Tajikistan OR Benin OR Burkina Faso OR (Burkina Faso or Burkina Fasso or Upper Volta) OR Burundi OR (Burundi or Ruanda‐Urundi) OR Central African Republic OR (Central African Republic or Ubangi‐Shari) OR Chad OR Democratic Republic Congo OR (((Democratic Republic or DR) adj2 Congo) or Congo‐Kinshasa or Belgian Congo or Zaire or Congo Free State) OR Eritrea OR Ethiopia OR Gambia OR Guinea OR Guinea‐Bissau OR (Guinea‐Bissau or Portuguese Guinea) OR Liberia OR Madagascar OR (Madagascar or Malagasy Republic) OR Malawi OR (Malawi or Nyasaland) OR Mali OR Mozambique OR Niger OR (Niger not (Aspergillus or Peptococcus or Schizothorax or Cruciferae or Gobius or Lasius or Agelastes or Melanosuchus or radish or Parastromateus or Orius or Apergillus or Parastromateus or Stomoxys) OR Rwanda OR (Rwanda or Ruanda) OR Sierra Leone OR Somalia OR south sudan OR South Sudan OR Tanzania OR (Tanzania or Tanganyika or Zanzibar) OR Togo OR (Togo or Togolese Republic or Togoland) OR Uganda OR Cambodia OR Indonesia OR Kiribati OR Laos OR (Laos or (Lao adj1 Democratic Republic)) OR “Federated States of Micronesia” OR Micronesia OR Mongolia OR Myanmar OR (Myanmar or Burma) OR Papua New Guinea OR (Papua New Guinea or German New Guinea or British New Guinea or Territory of Papua) OR Philippines OR Solomon Islands OR Timor‐Leste OR Timor‐Leste or East Timor or Portuguese Timor) OR Vanuatu OR (Vanuatu or New Hebrides) OR Viet Nam OR (Viet Nam or Vietnam or French Indochina) OR Kyrgyzstan OR (Kyrgyzstan or Kyrgyz Republic or Kirghizia or Kirghiz) OR Moldova OR exp. Ukraine OR exp. Uzbekistan OR Bolivia OR El Salvador OR Honduras OR Nicaragua OR Djibouti OR Egypt OR Morocco OR Tunisia OR Palestine OR (Gaza or West Bank or Palestine) OR Bangladesh OR Bhutan OR exp. India OR exp. Pakistan OR Angola OR Cameroon OR (Cameroon or Kamerun or Cameroun) OR Cape Verde OR (Cape Verde or Cabo Verde) OR Comoros OR (Comoros or Glorioso Islands or Mayotte) OR Congo OR (Congo not ((Democratic Republic adj3 Congo) or congo red or crimean‐congo)) OR Cote d'Ivoire OR (Cote d'Ivoire or Cote dIvoire or Ivory Coast) OR Eswatini OR (eSwatini or Swaziland) OR Ghana OR (Ghana or Gold Coast) OR Kenya OR (Kenya or East Africa Protectorate) OR Lesotho OR (Lesotho or Basutoland) OR Mauritania OR Nigeria OR “Sao tome and principe” OR (Sao Tome adj2 Principe) OR Senegal OR Sudan OR Zambia OR (Zambia or Northern Rhodesia) OR Zimbabwe OR (Zimbabwe or Southern Rhodesia) OR American Samoa OR china/ or guangxi/ or inner mongolia/ or macao/ or ningxia/ or tibet/ or Xinjiang OR China OR Fiji OR exp. Malaysia OR (Malaysia or Malayan Union or Malaya) OR Marshall islands OR Nauru OR Samoa OR ((Samoa not American Samoa) or Western Samoa or Navigator Islands or Samoan Islands) OR Thailand OR (Thailand or Siam) OR Tonga OR Tuvalu OR (Tuvalu or Ellice Islands) OR Albania OR Armenia OR exp. Azerbaijan OR Belarus OR (Belarus or Byelarus or Byelorussia or Belorussia) OR exp. “Bosnia and Herzegovina” OR (Bosnia or Herzegovina) OR Bulgaria OR exp. “Georgia (republic)” OR Georgia.ti,ab. not “georgia (u.s.)” OR Kazakhstan OR (Kazakhstan or Kazakh) OR Kosovo OR “Montenegro (republic)” OR Montenegro OR “republic of north macedonia” OR North Macedonia OR Romania OR exp. Russian Federation OR ussr OR (Russia or Russian Federation or USSR or Union of Soviet Socialist Republics or Soviet Union) OR exp. Serbia OR “Turkey (republic)” OR (Turkey.ti,ab. not “Turkey (bird)”/) or (Anatolia or Asia Minor) OR Turkmenistan OR Argentina OR (Argentina or Argentine Republic) OR Belize OR exp. Brazil OR Colombia OR Costa Rica OR Cuba OR Dominican Republic OR Ecuador OR Grenada OR Guatemala OR Guyana OR (Guyana or British Guiana) OR Jamaica OR exp. Mexico OR (Mexico or United Mexican States) OR Paraguay OR Peru OR Saint Lucia OR (St Lucia or Saint Lucia or Iyonala or Hewanorra) OR “Saint Vincent and the Grenadines” OR (Saint Vincent or St Vincent or Grenadines) OR Suriname OR (Suriname or Dutch Guiana) OR Venezuela OR Algeria OR Iran OR (Iran or Persia) OR exp. Iraq OR (Iraq or Mesopotamia) OR Jordan OR Lebanon OR (Lebanon or Lebanese Republic) OR Libyan Arab Jamahiriya OR Libya OR Maldives OR Sri Lanka OR (Sri Lanka or Ceylon) OR Botswana OR (Botswana or Bechuanaland or Kalahari) OR Equatorial Guinea OR (Equatorial Guinea or Spanish Guinea) OR Gabon OR (Gabon or Gabonese Republic) OR Mauritius OR (Mauritius or Agalega Islands) OR Namibia OR (Namibia or German South West Africa) OR South Africa OR (South Africa or Cape Colony or British Bechuanaland or Boer Republics or Zululand or Transvaal or Natalia Republic or Orange Free State)
Population (NDD)	(“attention deficit” or disruptive behavior disorders or ADHD or developmental disabilities or intellectual disability or learning disabilities) OR (Asperger Syndrome or Autistic Disorder or Autism Spectrum Disorder or ASD or Autis*) OR ((learning or developmental or intellectual) adj2 disability) OR (Disability or Learning disability or Developmental disabilities or neurod*) OR ((intellectual or learning or cognitive or mental Idiopathic) adj2 (impair* or disabilit* or disabl* or handicap*)) OR (Cerebral Pal* or motor disability) OR (Epilepsy or Epilepsy, Generalized or Epilepsy, Frontal Lobe or Epilepsy, Absence) OR fetal alcohol syndrome
Intervention	(school* adj2 (intervention* or program* or support or strategy or therap* or technique or treatment* or promotion* or prevention or service or inclus* or participat* or rehabilit* or mental health or counselling)) OR (“teachers training” or Peer support or Parents support).

Abbreviations: LMICs, low‐ and middle‐income countries; NDD, neurodevelopmental disorders.
